# Dynamic Changes in the Gut Microbiota and Metabolites during the Growth of Hainan Wenchang Chickens

**DOI:** 10.3390/ani13030348

**Published:** 2023-01-19

**Authors:** Yingzhi He, Jie Li, Feifan Wang, Wei Na, Zhen Tan

**Affiliations:** School of Animal Science and Technology, Hainan University, Haikou 570228, China

**Keywords:** Wenchang chickens, cecal microbiota, growth stage, metabolomic, 16S rRNA

## Abstract

**Simple Summary:**

From birth to adulthood, the large number of microbes that in-habit the gut play an important role in many aspects of the host’s normal physiological activities. Research on the development of gut microbiota and its metabolites in local chickens remains unclear. In this study, the cecal microbiota and metabolites in different developmental stages of Hainan Wenchang chickens (a native breed of Bantam) were investigated using 16S rRNA sequencing and untargeted metabolomics. With the increase of age, the gut microbiota tended to be more stable. Gut microbiota and their metabolites may have structural and functional changes in response to nutrient metabolism and immune requirements in different physiological states. The bacteria that form networks with their significant related metabolites were different in different developmental stages. These findings could potentially provide new insights into the physiological and molecular mechanisms of developmental changes of local chicken breed, as well as resources for microbial and metabolic biomarkers identification to improve the growth efficiency.

**Abstract:**

Gut microbiota and their metabolites play important roles in animal growth by influencing the host’s intake, storage, absorption, and utilization of nutrients. In addition to environmental factors, mainly diet, chicken breed and growth stage also affect changes in the gut microbiota. However, little research has been conducted on the development of gut microbiota and its metabolites in local chickens. In this study, the cecal microbiota and metabolites in different developmental stages of Hainan Wenchang chickens (a native breed of Bantam) were investigated using 16S rRNA sequencing and untargeted metabolomics. With aging, the structure of gut microbiota tended to be more stable. The relative proportions of dominant bacteria phyla Firmicutes, Bacteroidetes, and Proteobacteria showed stage changes with the development. With aging, gut microbiota and their metabolites may have structural and functional changes in response to nutrient metabolism and immune requirements in different physiological states. Several microbial and metabolic biomarkers with statistical differences were detected in different development stages. The bacteria that form networks with their significant related metabolites were different in various growth stages, including *uncultured_bacterium_f_Ruminococcaceae, Ruminococcaceae_UCG-014, Faecalibacterium, uncultured_bacterium_o_Bacteroidales,* and *uncultured_bacterium_f_Lachnospiraceae*. Partially differential bacteria were significantly correlated with short-chain fatty acids such as butyric acid. These findings may provide new insights into the physiological and molecular mechanisms of developmental changes of local chicken breeds, as well as resources for microbial and metabolic biomarker identification to improve growth efficiency.

## 1. Introduction

The health of the intestine is crucial for the growth and development and disease prevention of animals. From birth to adulthood, the large number of microbes that inhabit the gut play an important role in many aspects of the host’s normal physiological activities, including aiding nutrient absorption and digestion [1,2,3,4], energy consumption, storage and redistribution, immunity, and physiological health, to name a few, to maintain the body’s homeostasis at different growth stages [5]. At the same time, under the background of prohibiting the addition of antibiotics in feed, it is a general trend of antibiotic substitution production to promote the intestinal health of livestock and poultry by changing the structure of gut microbial composition and ensuring the growth and production efficiency of animals [6,7].

Eggs and meat of chickens are common sources of animal protein for humans; the healthy development of the chicken industry is important for animal husbandry. Chicken cecum has the highest microbial concentration, which affects the health and performance of the host. In recent years, several studies have revealed the structural characteristics of chicken gut microbiota through high-throughput sequencing [6,7,8,9,10,11,12,13,14], in particular cecal microbiota. The cecal microbiota of chickens is dominated by the phyla Bacteroidetes, Firmicutes, Proteobacteria, and Actinobacteria [8,15]. Host genetics and environment, especially feed, jointly shape the cecal microbiota. In addition to feed and other environmental factors [4,15], age is also an important factor affecting the cecal microbiota of laying hens. The cecal microbiota of young chickens is unstable and highly variable [16,17,18]. The core microbiota structure of the chicken gut tract is generally defined as at least 20-week-old adult chickens [19,20,21,22]. In the late egg-laying period, because of the decrease in gut microbiota richness, pathogenic bacteria are more likely to colonize in the gut [11].

The relative abundance of cecal microbiota in specific pathogen-free (SPF) chickens increased significantly with age, but the genera *Lactobacillus* and *Enterobacter* were opposite. At the age of 28 days, the normal microbial structure was basically established, and then became relatively stable at 42 days [23]. In the proximal digestive tract of adult chickens, *Lactobacillus* spp. is the dominant bacteria, and of course, there are some other species [24,25]. In the hindgut, the composition and complexity of the microbiota increased significantly with the continuation of the intestine [7]. However, within the same study, even if the study conditions are carefully controlled and intended to remain similar in each trial, there may be differences in the composition of intestinal microbiota in independent poultry studies [26,27].

The composition of cecal microbiota in broilers had unique structures at different developmental stages, but little is known on the relation between gut microbiota and development in local chicken breeds. Wenchang chickens, a native breed of Hainan, China, are the most economically important livestock sector in Hainan Province [28]. Wenchang chickens are famous for their coarse grain resistance, heat resistance, and excellent meat quality [29,30,31]. They have high intramuscular fat and moderate subcutaneous fat. Generally sold at about 160 days, chickens are raised in three stages: the chick-raising stage (1–30 days), the mountain forest free-range breeding stage (30–105 days), and the cage-fattening stage (105–160 days). The cecal microbial compositions before and after fattening were changed [32], and the change of gut microbiota might play an important role in each growth stage. Studying the gut microbial composition of Wenchang chickens and the developmental changes in metabolic function is helpful to improve the structure of the gut microbiota by means of intervention and improve the growth and production efficiency, and it has important practical significance for improving the competitiveness of Wenchang chicken products, and thus improving the economic benefits.

## 2. Materials and Methods

### 2.1. Animals, Phenotype Selection, and Sample Collection

All experimental Wenchang chickens were collected from a commercial farm of Tanniu Co., Ltd. in Hainan Province (Wenchang, China). The chickens were fed corn and soybean meal-based commercial diets (Supplementary Table S1) and water ad libitum under the same conditions, and no antibiotics were used during the study period. Samples were all female and collected at hatching (1 day old, 31.93 ± 1.85 g), brooding stage (27 days old, 185.57 ± 30.14 g), breeding stage (53 days old, 646.69 ± 74.22 g), fattening stage I (105 days old, 1187.08 ± 104.67 g), fattening stage II (133 days old, 1480.42 ± 90.06 g), and fattening stage III (161 days old, 1470.92 ± 94.29 g) (Supplementary Table S2). Chickens were raised in cages (50 cm × 50 cm × 50 cm, and 80 cm above ground). At each time point, 12 healthy female chickens were randomly selected and slaughtered by cervical dislocation. One of the samples in group d027 was distinguished from other samples within the group, then the sample was not selected in the analysis process, and the sample size in other groups was 12. The cecal sac was immediately opened with sterile scissors and the contents were recovered into a 2 mL centrifuge tube and snap-frozen in liquid nitrogen under aseptic conditions. All samples were transferred to −80 °C refrigerator for preservation until DNA extraction.

### 2.2. 16S rRNA Gene Sequencing and Analysis

Microbial genomic DNA was extracted from 71 cecal digesta samples using a QIAamp DNA Stool Mini Kit (Qiagen, Hilden, Germany) and following the manufacturer’s protocol. The V3–V4 region of the 16S rRNA gene was PCR-amplified using universal bacterial 16S rRNA gene PCR amplicon primers [33], 338F (5′-ACTCCTAGGAGAGGCAG CA-3′) and 806R (5′-GGCTACHVGGGTWTCTAT-3′). The thermocycling protocol was as follows: 98 °C for 1 min, followed by 28 cycles of 95 °C for 30 s, 55 °C for 30 s, and 72 °C for 30 s; and a final extension at 72 °C for 10 min. PCR products were purified and used to construct the libraries. The constructed libraries were sequenced using the Illumina Hiseq 2500 platform (2 × 250 paired ends). The bioinformatics analyses of the sequencing data were performed following the standard protocol [34]. The data were deposited in the National Center for Biotechnology Information’s Short Read Archive under the accession number SRP334838.

To minimize the effects of random sequencing errors, raw data files were filtered using Trimmomatic (version 0.33), merged using FLASH version 1.2.7 [35,36,37] and quality-controlled using UCHIME (version 4.2) [38]. Sequences with ≥97% similarity to obtain operational taxonomic units (OTUs) were clustered using USEARCH version 10.0 [39]. By default, 0.005% of all sequenced sequences were used as the threshold to filter OTUs. Representative sequences of OTUs were screened and compared with the 16S rRNA microbial reference database Silva Release 132 using the classification-consensus-BLAST method [40].

The Chao1 and Shannon indices were calculated by QIIME2 software to evaluate the alpha diversity of the samples [41]. Based on the distance matrix, the beta diversity per group was calculated using principal coordinate analysis [42] (PCoA), and PERMANOVA analysis was carried out using vegan package in R. Linear discriminant analysis (LDA) effect size (LEfSe) was used to analyze and identify biomarkers that differed significantly between groups [43]. Statistical significance was set at an LDA score ≥ 4 [44]. The software Phylogenetic Investigation of Communities by Reconstruction of Unobserved States 2 (PICRUSt2) was used to predict functional genes. The *p*-value threshold was 0.05, and functional pathways were assigned based on the Kyoto Encyclopedia of Genes and Genomes (KEGG) database [45].

### 2.3. Real-Time PCR

Quantitative real-time PCR tests were performed using ChamQ Universal SYBR qPCR Master Mix (Vazyme Biotech Co., Ltd., Nanjing, China) for bacteria with differences in LEfSe analysis. The universal 16S rRNA gene was used as the internal reference, and the sequences of all species-specific primers used are listed in Supplementary Table S3.

### 2.4. Untargeted Metabolomics Study and Data Analysis

The LC/MS system for metabolomics analysis is composed of Waters Acquity I-Class PLUS ultrahigh-performance liquid tandem Waters Xevo G2-XS QTof high-resolution mass spectrometer. The column used was purchased from Waters Acquity UPLC HSS T3 column (1.8 um 2.1 × 100 mm). Waters Xevo G2-XS QTOF high-resolution mass spectrometer can collect primary and secondary mass spectrometry data in MSe mode under the control of the acquisition software (MassLynx V4.2, Waters). In each data acquisition cycle, dual-channel data acquisition can be performed on both low collision energy and high collision energy at the same time. The low collision energy is 2 V, the high collision energy range is 10~40 V, and the scanning frequency is 0.2 s for a mass spectrum. The parameters of the ESI ion source are as follows: capillary voltage: 2000 V (positive ion mode) or —1500 V (negative ion mode); cone voltage: 30 V; ion source temperature: 150 °C; desolvent gas temperature 500 °C; backflush gas flow rate: 50 L/h; desolventizing gas flow rate: 800 L/h [46].

The raw data collected using MassLynx V4.2 were processed by Progenesis QI software for peak extraction, peak alignment, and other data-processing operations, based on the Progenesis QI software online METLIN database and Biomark’s self-built library for identification, and at the same time, theoretical fragment identification and mass deviation all are within 100 ppm. Principal component analysis and Spearman correlation analysis were used to judge the repeatability of the samples within group and the quality control samples. The identified compounds were searched for classification and pathway information in Kyoto Encyclopedia of Genes and Genomes (KEGG), Human Metabolome Database (HMDB), and lipidmaps databases [47].

### 2.5. Statistical Analysis

Data are presented as the means ± standard error. SPSS 21.0 software (SPSS, Chicago, IL, USA) was used for one-way ANOVA of body weight and quantitative real-time PCR, and Tukey’s test was used to confirm significant difference between groups. The software GraphPad Prism v8.0.2 (Motulsky, Los Angeles, CA, USA) was used for statistical analysis and mapping of quantitative real-time PCR results. According to the grouping information, the difference multiples were calculated and compared; T test was used to calculate the difference significance *p* value of each compound. The R language package ropls was used to perform OPLS-DA modeling, and 200 times permutation tests were performed to verify the reliability of the model. The VIP value of the model was calculated using multiple cross-validation. The method of combining the difference multiple, the *p* value, and the VIP value of the OPLS-DA model was adopted to screen the differential metabolites. The screening criteria are FC > *p* value 1. The difference metabolites of KEGG pathway enrichment significance were calculated using hypergeometric distribution test. Correlation between microbiota and metabolites was performed by Spearman correlation analysis with a threshold of correlation coefficient of 0.8, and the *p* value of correlation was 0.05 [48].

## 3. Results

### 3.1. Microbial Composition at Different Developmental Stages in Wenchang Chickens

The body weight of Wenchang chickens changed with age and increased gradually from hatching to 133 days old (*p* < 0.01). Except for the comparison between the 133-day and 161-day groups, the difference between the other stages was extremely significant (Supplementary Table S2). By analyzing 71 samples from hatching to 161 days old, we obtained 5,664,380 effective reads, with an average of 79,780 high-quality reads per sample (Supplementary Table S3). A total of 753 OTUs at 97% identity were detected, 749 of which were shared among all groups (Supplementary Figure S1). Three OTUs were unique in the 1-day age group, while one OTU was unique in the 105-day age group.

**Figure 1:**
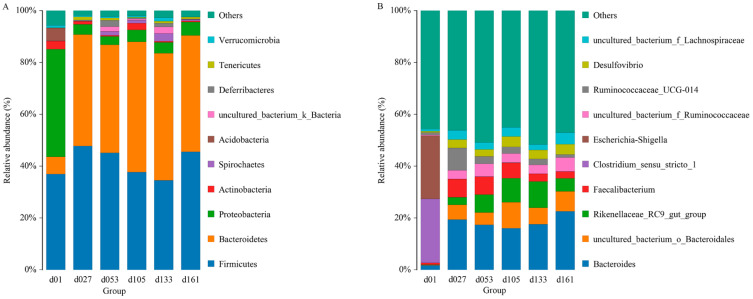
Top ten of the microbial composition at the phylum level (**A**) and genus level (**B**). Each bar represents the average relative abundance of each taxon within a time point. d01, *n* = 12; d027, *n* = 11; d053, *n* = 12; d0105, *n* = 12; d133, *n* = 12; d161, *n* = 12.

From a taxonomic perspective, 23 phyla, 40 classes, 80 orders, 135 families, 267 genera, and 301 species were identified in the cecal samples of all 71 chickens. Proteobacteria, Bacteroidetes, and Firmicutes were the major phyla during the development of Wenchang chickens, accounting for more than 70% of all OTUs (Figure 1A). The relative abundance of Proteobateria and Bacteroidetes in group d01 was obviously different from that in other days. In group d01, the relative abundance of Proteobasteria was 41.54%, that of Bacteroidetes was 6.62%, while Acidobasteria was 4.97%, and other groups were lower than 0.07%. After feeding, Proteobateria in d27 group decreased to 3.99%, and remained below 6% in the later-days age groups, while the abundance of Bacteroidetes increased to 42.95%. From the d53 group, the relative abundance of Spirochaetes was higher than 1%, while in d01 (0.08%) and d27 (0.004%) groups, the abundances were both less than 0.1%.

At the genus level, the relative abundance of *Clostridium_ sensu_ stricto_1* and *Escherichia-Shigella* were 24.66% and 24.36% in group d01, respectively, and both were lower than 0.2% in other groups (Figure 1B). The proportions of *Faecalibacterium* in group d01, d27, d53, d105, d133, and d161 were 0.67%, 7.02%, 6.91%, 5.90%, 2.95%, and 2.66%, respectively, and the relative abundance of *Bacteroides* increased from 1.76% of 01d to 19.40% (d27), and then remained higher than 16%. The proportion of *Ruminococcaceae_UCG-014* increased from 0.98% of 01d to 8.65% of d27, and then gradually decreased to 1.21% of d161.

### 3.2. Microbial Diversity at Different Developmental Stages in Wenchang Chickens

**Figure 2:**
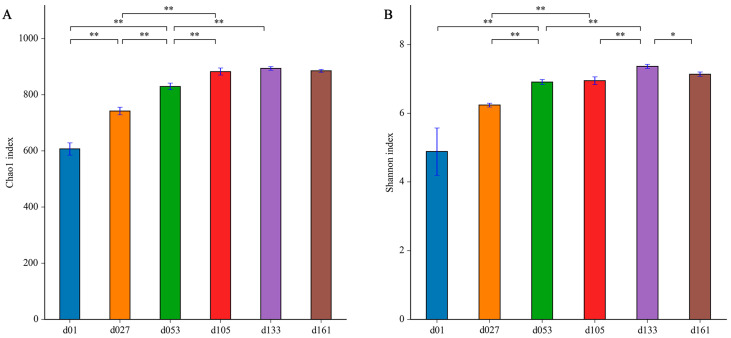
Dynamic changes of cecal microbiota alpha diversity with age in Wenchang chickens. (**A**) Chao1 index; (**B**) Shannon index. Different letters represent significant differences in alpha diversity indices based on Student’s *t*-test (* *p* < 0.05, ** *p* < 0.01). d01, *n* = 12; d027, *n* = 11; d053, *n* = 12; d0105, *n* = 12; d133, *n* = 12; d161, *n* = 12.

To characterize the levels and patterns of diversity within individuals, the alpha diversity indices of the cecal microbial structure of the six groups were compared. The values of Chao1 and Shannon both continuously increased (Figure 2). The lowest microbial diversity was observed at 1 day old, which was significantly lower than that of other groups (*p* < 0.05). From hatching to 27 days old, the microbial diversity was significantly improved (*p* < 0.05), and the diversity increased with development and tended to be stable after the second fattening stage (d133).

**Figure 3:**
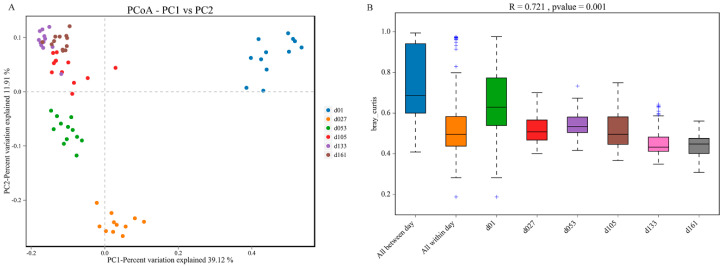
Dynamic changes of cecal microbiota beta diversity with age in Wenchang chickens. (**A**) Principal coordinates analysis (PCoA) based on Bray-Jaccard method. (**B**) Anosim based on the Bray-Curtis method. d01, *n* = 12; d027, *n* = 11; d053, *n* = 12; d0105, *n* = 12; d133, *n* = 12; d161, *n* = 12.

Principal coordinate analysis (PCoA) based on weighted UniFrac distances was performed, and revealed clear clustering of cecal bacterial communities between groups (Figure 3). All the samples were divided into four major clusters: the samples of group d01, group d27, and group d53 were clustered independently and divided into three clusters, and the samples of group d105, d133, and d161 were clustered together and formed the fourth cluster. Starting from group d01, the distance of each group gradually shortened with the increase in age, and the sample similarity gradually increased (Figure 3A). Significant differences existed between the samples of d01, d27, and d53 groups and those of other groups. In PERMANOVA analysis, the *p* value was less than 0.05, while the R value was greater than 0.7, indicating that the difference between groups was greater than the difference within groups, and the difference between groups gradually narrowed with the increase in age (Figure 3B).

**Figure 4:**
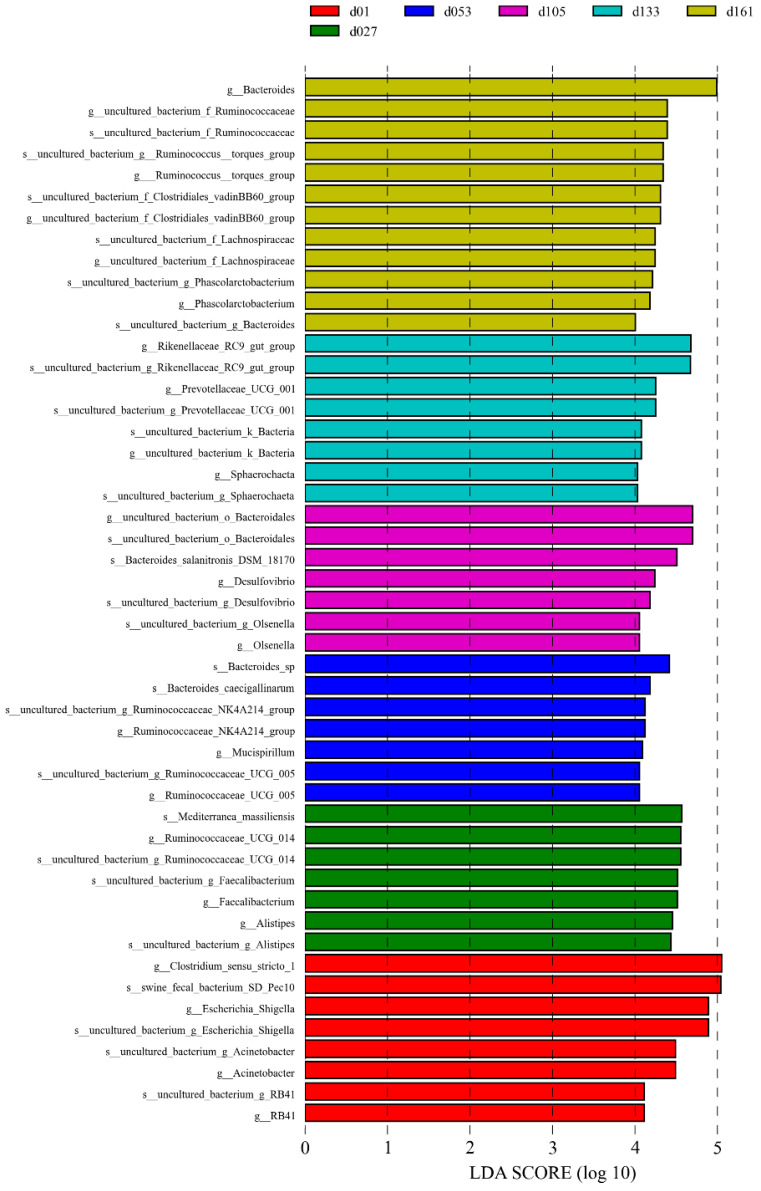
Cecal microbiota differences of Wenchang chickens at different development stages. A histogram of linear discriminant analysis (LDA) scores computed for differences in the proportions of cecal microbiota. Taxa meeting an LDA significant threshold of > 4 are shown. d01, *n* = 12; d027, *n* = 11; d053, *n* = 12; d0105, *n* = 12; d133, *n* = 12; d161, *n* = 12.

The significant biomarkers among the different groups were analyzed using linear discriminant analysis effect size (LEfSe); the LDA score was greater than 4.0 (Figure 4). A total of 49 biomarkers were statistically different, with 12 in the d01 group, 8 in the d27 group, 7 in the d53 group, 7 in the d105 group, 7 in the d133 group, and 8 in the d161 group. The genera *Escherichia-Shigella* and *Acinetobacter* were significantly enriched in the d01 group, and *Faecalibacterium* was significantly enriched at 27 days old. At 53 days of age, *Ruminocochaceae_ UCG_ 005* was enriched, Desulfovibrio was significantly enriched at 105 days old, while *Rikenellaceae_RC9_gut_group* and Prevotellaceae_UCG_001 were significantly enriched at 133 days of age and Bacteroides was enriched at 161 days of age. Meanwhile, the comparative analysis between two pairs showed 32 microbial biomarkers at the age of hatching to 27 days, 13 at the age of 27 to 53 days, 6 at the age of 53 to 105 days, 3 at the age of 105 to 133 days, and 5 biomarkers from 133 to 161 days old (Supplementary Figure S2). Among them, from 27 to 53 days old, *Lactobacillus* was significantly enriched in the d27 group and *uncultured_bacterium_f_Prevotellaceae* was significantly enriched in the d53 group. The comparison from 53 days to 161 days showed that except for the high abundance of *Mucispirillum* in the d53 group, the significantly enriched genera in other groups were mainly Bacteroides, *Rikenellaceae_RC9_gut_group*, and *Ruminococcus_torques_group*. These differences were similar to the results verified by quantitative real-time PCR of bacteria genera. The relative proportion of *Lactobacillus* in the d27 group was significantly higher than that of the d53 group, and the relative proportion of *Faecalibacterium* gradually decreased with the increase in age (Student’s *t*-test, *p* < 0.05, Supplementary Figure S3).

### 3.3. Microbial Functional Prediction at Different Developmental Stages in Wenchang Chickens

**Figure 5:**
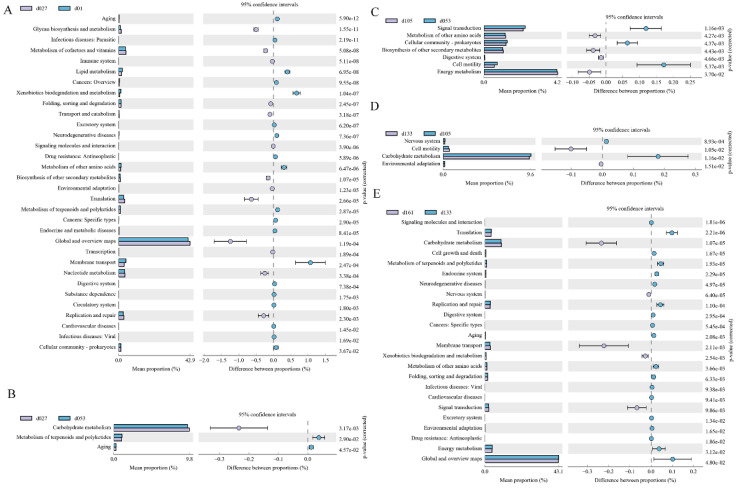
Enrichment of KEGG metabolic pathways of the cecal microbial community at different developmental stages of Wenchang chickens. (**A**) Functional pathways that changed significantly (*p* < 0.05) between d01 and d027. (**B**) d027 vs. d053, (**C**) d053 vs. d105, (**D**) d105 vs. d133, (**E**) d133 vs. d161. d01, *n* = 12; d027, *n* = 11; d053, *n* = 12; d0105, *n* = 12; d133, *n* = 12; d161, *n* = 12.

The second-level KEGG pathways were verified between two adjacent growth stages, respectively (Figure 5). Among them, 32 KEGG pathways were significantly different between hatching and 27 days of age (*p* < 0.05). In the d01 group, 19 pathways, including parasitic, xenobiotic biodegradation and metabolism, and membrane transport, were significantly enriched, while 13 pathways, including glycan biosynthesis and metabolism, global and overview maps, were enriched in the d27 group (Figure 5A). Only three pathways were different between the d27 and d53 groups (*p* < 0.05), and the enrichment of carbohydrate metabolism in the d27 group compared with the d53 group (Figure 5B). In the comparison between the d53 and d105 groups, seven pathways were different (*p* < 0.05), and signal transmission, cellular community prokaryotes, and cell mobility were significantly enriched in group d53, while metabolism of other amino acids, digestive system, energy metabolism, and biosynthesis of other secondary metals were significantly enriched in group d105 (Figure 5C). Between the d105 and d133 groups, four pathways were detected to be different (*p* < 0.05). The nervous system and carbohydrate metabolism were enriched in the d105 group, while cell mobility and environmental adaptation were enriched in the d133 group (Figure 5D). A total of 24 metabolic pathways between the d133 group and d161 group, and 5 pathways, including carbohydrate metabolism, endocrine system, and signal transformation, were significantly enriched in the d161 group, while other metabolic pathways were enriched in the d133 group (*p* < 0.05) (Figure 5E).

Under the second classification level, COG function comparison notes were verified (Supplementary Figure S4). From hatching to 27 days old, 17 functions were differently enriched (*p* < 0.05). Nine terms, including inorganic ion transport and metabolism, secondary metals biosynthesis, transport, and catalysis, were significantly enriched in the d01 group, while eight functions were enriched in the d27 group (*p* < 0.05) (Supplementary Figure S4A). Six terms were annotated between the d27 and d53 groups (*p* < 0.05), carbohydrate transport and metabolism was higher in the d27 group, while lipid transport and metabolism was enriched in the d53 group (Supplementary Figure S4B). Results of other comparisons were shown in Supplementary Figure S4C–E.

### 3.4. Metabolite Characteristics at Different Developmental Stages in Wenchang Chickens

The metabolic characteristics of cecal contents in different age groups were detected by LC/MS, and principal component analysis (PCA) was performed to describe metabolic differences between samples (Supplementary Figure S5). Samples in different days’ age groups were relatively clustered and separated among groups. To further explore the effect of development stages on metabolites in the cecal microbiota, orthogonal projections to latent structures-discriminant analysis (OPLS-DA) was performed. The lowest values of R2Y and Q2Y were 0.998 and 0.571, respectively, indicating that the model built by the OPLS-DA method could distinguish the correct sample group by metabolic expression. At the significance threshold of VIP > 1, *p* < 0.05, and FC = 1, pairs of d27 vs. d53, d53 vs. d105, d105 vs. d133, and d133 vs. d161 were carried out, respectively. In the positive ion model, 98 metabolites were upregulated and 70 metabolites were downregulated in d27 vs. d53. A total of 248 (135 upregulated and 113 downregulated) in d53 vs. d105, 362 (173 upregulated and 189 downregulated) in d105 vs. d133, and 224 (106 upregulated and 118 downregulated) in d133 vs. d161 differential metabolites were screened. In the negative ion mode, 26 metabolites were upregulated and 59 metabolites were downregulated in d27 vs. d53. A total of 101 (68 upregulated and 33 downregulated) in d53 vs. d105, 145 (78 upregulated and 67 downregulated) in d105 vs. d133, and 136 (63 upregulated and 73 downregulated) metabolites were differential screened in d133 vs. d161.

All metabolites identified were assigned to the KEGG databases. The pathway biosynthesis of plant secondary metabolites (ko01060) was significantly different between 27 and 53 days of age (*p* < 0.05). In d53 vs. d105, porphyrin metabolism (ko00860) and toluene degradation (ko00623) were significant enrichment pathways. The significant differences between 105-day-old and 133-day-old pathways were concentrated in drug metabolism-cytochrome P450(ko00982), neomycin, kanamycin and gentamicin biosynthesis (ko00524), ABC transporters (ko02010), aminoacyl-tRNA biosynthesis (ko00970), mineral absorption (ko04978), D-Amino acid metabolism (ko00470), protein digestion and absorption (ko04974), and monobactam biosynthesis (ko00261), which were significantly different between d133 and d161 groups (*p* < 0.05).

### 3.5. Correlation Analysis between Microbiota and Metabolites

**Figure 6:**
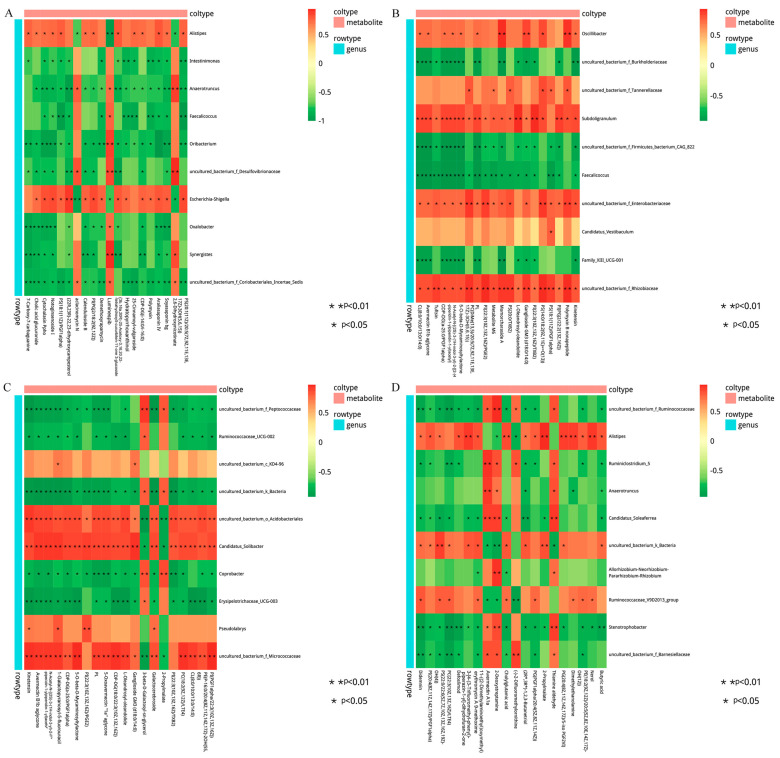
Heatmap of the correlation between differential cecal microbes and metabolites. The different colors represent the magnitude of spearman’s correlation coefficient. Red color indicates positive correlation, while green color indicates negative correlation. * *p* < 0.05, ** *p* < 0.01. Heat map of top 20 differential genus and top 20 differential metabolite correlations: (**A**) d27 vs. d53, (**B**) d53 vs. d105, (**C**) d105 vs. d133, (**D**) d133 vs. d161. d027, *n* = 5; d053, *n* = 5; d0105, *n* = 5; d133, *n* = 5; d161, *n* = 5.

**Figure 7:**
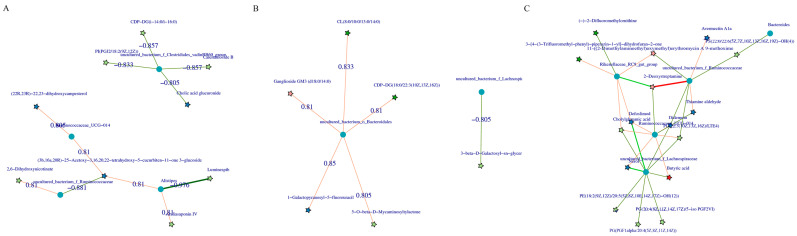
Correlation network diagram of the top 20 significant microbe–metabolite pairs. Pentagrams represent metabolites and circles represent microbes. The red line represents a positive correlation, the green line represents a negative correlation, and the wider the line, the higher the correlation, (**A**) correlation network diagram of all metabolites with all microorganisms (d27 vs. d53), (**B**) d105 vs. d133, (**C**) d133 vs. d161. d027, *n* = 5; d053, *n* = 5; d0105, *n* = 5; d133, *n* = 5; d161, *n* = 5.

The correlation between the metabolites and the relative abundance of cecal bacteria between two adjacent growth stages were assessed, respectively. At the comparisons of different development stages, the correlations of the top different bacteria (phylum or genus level) with their associated metabolites were varied (Figure 6), and the strength of correlation during 0–0.20 was negligible, 0.21–0.35 was weak, 0.36–0.67 was moderate, 0.68–0.90 was strong, and 0.91–1.00 was very strong. Most of the microorganisms had the same trend of association with the metabolites, but only a few did not. For example, *Alistipes* and *Escherichia-Shigella* were positively correlated with several metabolites and negatively correlated with three metabolites (2, 6-dihydroxynicotinate, Luminespib, and aclacinomycin N) in d27 vs. d53 groups. Meanwhile, these three metabolites were positively correlated with other microorganisms, and some of them were significantly positively correlated. A similar phenomenon was observed in the comparisons of other groups (Figure 6B–D). In order to further demonstrate and understand the association between microbiota and metabolites, the correlation network of the top 20 different microorganisms and metabolites were constructed (Figure 7). Overall, these findings agreed with the observed taxa enrichment and the presence of metabolites in the cecum.

## 4. Discussion

In this study, Wenchang chickens were raised from hatching to 105 days of age; then, in order to further enhance the flavor, fat content, and quality of the meat, the chickens were fattened eight weeks before marketing, until 161 days. The body weight did not increase significantly after 133 days of age (Supplementary Table S2), indicating that the body weight stabilized and the samples could fully cover and represent the whole growth cycle of Wenchang chickens. Alpha diversity increased rapidly from the 1st to 27th day of age and increased with development, and then tended to be stable after 105 days of age, which is similar to previous studies [49]. The gut microbiota of chickens was immature in the early stage, and the diversity of microbiota was low. The microbial structure was different from that of other growth stages; with the increase in age, a stable structure was formed. Firmicutes, Bacteroidetes, and Proteobacteria were all dominant phyla at all development stages, accounting for more than 70% of total bacteria community, which was consistent with previous studies [50].

In recent years, studies have shown that the vagina contains a very complex microbial community, which might be transmitted to the fetus through the mother [51]. Hens could vertically transmit gut microbes to embryos through fertilization and egg formation in the oviduct [52]. Affected by vertical transmission from hens, the composition of gut microbes in newly hatched chicks may be more similar to those in the hen upper reproductive tract or chicken embryos. Here, the proportion of Proteobacteria was as high as 41.54% at the 1st day of age, and the abundance of Bacteroidetes was low, which was similar to previous studies [53]. Different from Firmicutes, which kept a high abundance from the beginning, Bacteroidetes only reached 6.62% at the age of 1 day and 42.95% at the age of 27 days, mainly due to the introduction of feed. Bacteroidetes contained a variety of polysaccharide utilization loci sites (PULs), which could decompose cellulose, carbohydrates, and various polysaccharides, and was closely related to the metabolism of the host [54]. From birth to one month of age, the relative abundance of Bacteroides in cecum of Wenchang chickens changed from low to high. At this stage, accompanied by the growth and development, environmental factors, especially dietary conditions (feed composition) greatly affected the colonization of Bacteroides. Bacteroidetes mainly produce acetic acid and propionate, while Firmicutes produce more butyrate. A high proportion of Firmicutes/Bacteroidetes could lead to obesity, because Firmicutes could extract energy from feed more effectively than Bacteroidetes. Thus, the intestinal tract was promoted to absorb energy more efficiently [55,56]. In this study, the proportion of Firmicutes/Bacteroidetes decreased with the increase in age from 1 to 133 days old, and gradually decreased from 5.58 to 0.70, consistent with the previous study where the proportion of Firmicutes/Bacteroidetes in Dagu chickens decreased from 12 weeks to 18 weeks [57].

At the genus level, the relative abundance of *Escherichia-Shigella* and *Acinetobacter* at 1 day of age was significantly higher than that of the other groups, which was due to the imperfect development of the immune system of chicks. Exposed to the environment after hatching, they were susceptible to invasion by harmful microorganisms from the environment, such as eggshell-borne feces, sewage, and the feces of the chicks themselves. *Escherichia-Shigella* and *Acinetobacter* were both conditional pathogens that could cause long-term intestinal inflammation in dysentery, acute diarrhea, and chronic diarrhea. Studies have proved that the addition of *Enterococcus faecium* can reduce the relative proportion of harmful bacteria such as *Escherichia* [58].

The relative abundance of *Bacteroides*, *Faecalibacterium*, and *Ruminiclostridium* were higher at 27 days of age compared with the 1st day. These genera are associated with higher productivity and immune status, and are often used as indicators of intestinal health of poultry [59]. *Faecalibacterium* is one of the important producers of butyric acid in the animal intestinal tract, and *Faecalibacterium prausnitzii* has been proven to be anti-inflammatory, maintain bacterial enzyme activity, and protect the digestive system from intestinal pathogens [60]. Short-chain fatty acids (SCFA) can improve intestinal health by maintaining the anaerobic intestinal environment and preventing the propagation of facultative anaerobic pathogens, and have an important impact on host physiology and energy balance [61,62]. *Ruminiclostridium* has the ability to degrade complex carbohydrates and may contribute to the degradation of dietary fiber [63]. The period of 1–27 days of age was the most vigorous period for the competitive replacement of carbohydrate metabolism and immune-related microbiota, and good production potential and good immune characteristics were shown at 27 days of age. In addition, *Lactobacillus* was also enriched at 27 days of age. The application of the probiotic *Lactobacillus* to poultry could promote growth; the effect is similar to that of antibiotics [64], and reduces gastrointestinal colonization by human food-borne pathogens, such as *campylobacter* [65] and *salmonella* [66]. At 53 days of age, *uncultured_bacterium_f_Prevotellaceae* and *Rikenellaceae_RC9_gut_group* were significantly higher than those at 27 days of age. *Prevotellaceae* is closely related to obesity and lipid metabolism [67], and *Prevotella* can utilize SCFAs, especially acetate, to affect the ecosystem composition and function of the intestinal microbiome, and enhance the sensitivity of the host to mucosal inflammation [68]. *Rikenellaceae_RC9_gut_group* has the potential to degrade polysaccharides and contribute to the digestion of water-soluble fibers [69]. From 27 to 53 days of age was an important stage in the growth and development of Wenchang chickens. This might be due to the needs of growth and development, in addition to the absorption and utilization of carbohydrates, the gut microbiota related to lipid metabolism was further enriched, and more different kinds of polysaccharides degrading bacteria began to colonize in the hindgut.

Compared with the 105-day-old group, *Mucispirillum* was enriched at 53 days old. *Mucispirillum* was positively correlated with fat metabolism such as free fatty acids and LysoPC (20:3) [70]. *Mucispirillum schaedleri* might compete with *Salmonella* to antagonize virulence and help the host resist colitis [71]. At present, the most troublesome of poultry products are the zoonotic diseases caused by *Campylobacter* and *Salmonella*. Therefore, understanding the antagonistic relationship between microorganisms is particularly important. The difference between 53 and 105 days of age is mainly different types of *Bacteroides*. *Uncultured_bacterium* and *Faecalibacterium* were found between 105 and 133 days of age. Again, the composition of cecal microbiota tended to mature from 105 days of age. Diseases, environmental information processing, lipid metabolism, secondary metabolite biosynthesis, transport and catabolism, and related microbial functions are enriched on the 1st day in Wenchang chickens. Chicks need to resist and adapt to the pressure from the environment at birth, and use the remaining yolk of the chick embryo after hatching for nutrient metabolism. Lipid- and protein-related metabolism of the host may affect the early microbial composition from the chick embryo to the shell breaking. Genetic information processing was significantly higher at the 27-day-old group than the 1-day-old group. From 1 to 27 days of age was the rapid growth and development stage of chickens.

Both KEGG analysis and COG function indicated that the carbohydrate-related metabolism was significantly enriched in the d27 group compared with the d53 group. During the growing stage, chickens kept ingesting nutrients in the feed, especially the requirements for carbohydrate digestion and energy conversion. Gut microbes related to carbohydrate metabolism might be able to multiply, which helps the host to better absorb and convert energy. Lipid transport and metabolism, protein turnover, and the metabolism of terpenoids and polyketides were enriched in the d53 group, which might be closely related to the muscle development, adipogenesis, and hormone synthesis and secretion of the host at this stage. Lipid metabolism was not significant different between the d53 and d105 groups, yet energy metabolism and the digestive system were enriched in the d105 group. Amino acid transport and metabolism, coenzyme transport and metabolism, and cellular processes were significant enriched in the d133 group compared with the d105 group. During this stage, body weight still increased, and different microbial functions might be related more to protein accumulation of the host. Body weight was not significantly different between 133 days of age and 161 days of age; the microbiota had few differences in microbial species but great differences in microbial functions, which also suggested that the same kinds of microorganisms might have different microbial functions at different stages, and help the host to complete different types of metabolism. In addition, with the maturation of gut microbiota, the composition became more similar between individuals, the little changes in the composition of the microbiota did not always lead to changes in the performance of the host [72,73].

The correlation analysis of cecal microbiota and its metabolites was preformed to reveal the effects of development stages on the gut of chickens. The bacteria that form networks with their significant related metabolites were different in different developmental stages, including *uncultured_bacterium_f_Ruminococcaceae, Ruminococcaceae_UCG-014, Faecalibacterium, uncultured_bacterium_o_Bacteroidales,* and *uncultured_bacterium_f_Lachnospiraceae.* Partially differential bacteria were significantly related to short-chain fatty acids (SCFAs), which provide up to 10% of the metabolizable energy in chickens [74]. A member of the SCFAs, butyric acid, maintains the integrity of the intestinal barrier by providing energy to the intestinal cells, regulates the balance of the gut microbiota by acidifying the intestinal environment, and affects intestinal immune function by inhibiting the migration of immune cells and regulating cell proliferation and apoptosis [75,76]. Gut microbiota may play an important role in multiple physiological processes of the organism through their metabolites, providing energy, activating immune cells, regulating lipid deposition, maintaining the balance of gut microbiota, and protecting intestinal health [77,78]. Metabolites of dysregulated microbiota may have negative effects on the host, such as involvement in the pathogenesis of tibial chondrodysplasia in chickens [79].

## 5. Conclusions

The present study showed differences in the cecal microbiota and metabolites from birth to market time of a local breed, Hainan Wenchang chickens. With the increase in age, the gut microbiota tended to be more stable. The relative proportions of dominant bacteria Firmicutes, Bacteroidetes, and Proteobacteria showed stage changes with the development. With aging, gut microbiota and their metabolites may have structural and functional changes in response to nutrient metabolism and immune requirements in different physiological states. Several microbial and metabolic biomarkers with statistical differences were detected in different development stages. These findings of dynamic changes in the cecal microbiota and metabolites during the growth could potentially provide new insights into the physiological and molecular mechanisms of developmental changes of local chicken breeds, as well as resources for microbial and metabolic biomarker identification to improve the growth efficiency.

## Data Availability

The data were deposited in the National Center for Biotechnology Information’s Short Read Archive BioProject repository, under the accession number PRJNA904554.

## Supplementary Materials

The following are available online at https://www.mdpi.com/article/10.3390/ani13030348/s1. Supplementary Table S1: Diet ingredient for chickens. The premix provided the following per kg of the diet: VA 12500 IU, VD_3_ 3500 IU, VE 25 mg, VB_1_ 3 mg, VB_2_ 8 mg, VB_6_ 8 mg, VB_12_ 0.03 mg, D-pantothenic acid 20 mg, niacin 60 mg, biotin 0.18 mg, folic acid 1.5 mg, Cu (as copper sulfate) 8 mg, Fe (as ferrous sulfate) 100 mg, Mn (as manganese sulfate) 100 mg, Zn (as zinc sulfate) 100 mg, I (as potassium iodide) 0.6 mg, Se (as sodium selenite) 0.16 mg. Supplementary Table S2: Weight table of Wenchang chickens by age. The same index and different groups of data with different uppercase letters indicate that the difference is extremely significant (*p* < 0.01). Supplementary Table S3: Sequences of specific gene primers for quantitative real-time PCR. Supplementary Table S4: Statistics of sample sequencing data processing results. Supplementary Figure S1: Shared OTU analysis of the different groups. d01, d027, d053, d105, d0133, d161, cecal microbiota of Wenchang chickens aged 1 day, 27 days, 53 days, 105 days, 133 days, 161 days, respectively. Supplementary Figure S2: Paired comparison of cecal microbiota of Wenchang chickens at different development stages. A histogram of linear discriminant analysis (LDA) scores computed for differences in the proportions of cecal microbiota. Taxa meeting an LDA significant threshold of >4 were shown. (A) d01 vs. d27, (B) d27 vs. d53, (C) d53 vs. d105, (D) d105 vs. d133, (E) d133 vs. d161. Supplementary Figure S3: Quantitative real-time PCR of cecal microorganisms. (A) Changes in relative abundance of *Lactobacillus* and verification chart, (B) changes in relative abundance of *Faecalibacterium* and verification chart. (* *p* < 0.05, ** *p* < 0.01). Supplementary Figure S4: Enrichment of COG functions of the cecal microbial community at different developmental stages of Wenchang chickens. (A) Functional pathways that changed significantly (*p* < 0.05) between d01 and d27. (B) d27 vs. d53, (C) d53 vs. d105, (D) d105 vs. d133, (E) d133 vs. d161. Supplementary Figure S5: Principal component analysis (PCA) based on metabolic differences between samples at different development stages. (A) Positive ion mode, (B) negative ion mode.
