# Supplementary material for: Dynamic Changes in the Gut Microbiota and Metabolites during the Growth of Hainan Wenchang Chickens
Source: Animals (Basel). 2023 Jan 19;13(3):348. doi: 10.3390/ani13030348 (PMC9913245; doi:10.3390/ani13030348)
Supplement: Supplementary file 1 [file animals-13-00348-s001.zip › Supplementary Table S1.pdf]

Supplementary Table S1 Diet ingredient for chickens.

Items	Diet
**Ingredients（%)**	
Corn	65
Soybean meal	24
Wheat bran	3
Peanut meal	4
Premix	4
Total	100
**Nutrient levels**	
Metabolic energy (MJ/kg)	12.18
Crude protein (%)	17.65
Ca (%)	0.4
Available P (%)	0.5
Lys (%)	0.88
Met (%)	0.38

The premix provided the following per kg of the diet: VA 12500 IU, VD_3 3500 IU, VE 25 mg, VB_1 3 mg, VB_2 8 mg, VB_6 8 mg, VB_{12} 0.03 mg, D-pantothenic acid 20 mg, niacin 60 mg, biotin 0.18 mg, folic acid 1.5 mg, Cu (as copper sulfate) 8 mg, Fe (as ferrous sulfate) 100 mg, Mn (as manganese sulfate) 100 mg, Zn (as zinc sulfate) 100 mg, I (as potassium iodide) 0.6 mg, Se (as sodium selenite) 0.16 mg.
