# Supplementary material for: Dynamic Changes in the Gut Microbiota and Metabolites during the Growth of Hainan Wenchang Chickens
Source: Animals (Basel). 2023 Jan 19;13(3):348. doi: 10.3390/ani13030348 (PMC9913245; doi:10.3390/ani13030348)
Supplement: Supplementary file 1 [file animals-13-00348-s001.zip › Supplementary Table S2.pdf]

Supplementary Table S2 Weight table of Wenchang chickens by age.

Age	Body weight(g)±SD
1day	31.93 ± 1.85^{E}
27day	185.57 ± 30.14^{D}
53day	646.69 ± 74.22^{C}
105day	1187.08 ± 104.67^{B}
133day	1480.42 ± 90.06^{A}
161day	1470.92 ± 94.29^{A}

The same index and different groups of data with different uppercase letters indicate that the difference is extremely significant ($p < 0.01$).
