# Supplementary material for: Dynamic Changes in the Gut Microbiota and Metabolites during the Growth of Hainan Wenchang Chickens
Source: Animals (Basel). 2023 Jan 19;13(3):348. doi: 10.3390/ani13030348 (PMC9913245; doi:10.3390/ani13030348)
Supplement: Supplementary file 1 [file animals-13-00348-s001.zip › Supplementary Table S3.pdf]

Supplementary Table S3 Sequences of specific gene primers for quantitative real-time PCR.

Target	PCR Product (bp)	Sense and Anti-sense primer	Reference
Lactobacillus	163	F:5'CCCTAAAGACTGGGATAC CAC3' R:5'TACGCATCATTGCCTTGG3'	[1]
Total bacteria		F:5'CGGTGAATACGTTCYCGG3' R:5' GGWTACCTTGTTACGACTT3'	[2]
Faecalibacterium	172	F :5'GGCGGGAGAGCAAGTCA GTG3' R:5'CGTCAGTTAGACCCCAGC AA3'	[3]

1. Zhan, Q.; Qi, X.; Weng, R.; Xi, F.; Chen, Y.; Wang, Y.; Hu, W.; Zhao, B.; Luo, Q. Alterations of the Human Gut Microbiota in Intrahepatic Cholestasis of Pregnancy. *Front Cell Infect Microbiol* **2021**, *11*, 635680, doi:10.3389/fcimb.2021.635680.
2. Suzuki, M.T.; Taylor, L.T.; DeLong, E.F. Quantitative analysis of small-subunit rRNA genes in mixed microbial populations via 5'-nuclease assays. *Applied and environmental microbiology* **2000**, *66*, 4605-4614, doi:10.1128/AEM.66.11.4605-4614.2000.
3. Duan, C.; Cui, Y.; Zhao, Y.; Zhai, J.; Zhang, B.; Zhang, K.; Sun, D.; Chen, H. Evaluation of Faecalibacterium 16S rDNA genetic markers for accurate identification of swine faecal waste by quantitative PCR. *J Environ Manage* **2016**, *181*, 193-200, doi:10.1016/j.jenvman.2016.06.022.
