# Supplementary material for: Dynamic Changes in the Gut Microbiota and Metabolites during the Growth of Hainan Wenchang Chickens
Source: Animals (Basel). 2023 Jan 19;13(3):348. doi: 10.3390/ani13030348 (PMC9913245; doi:10.3390/ani13030348)
Supplement: Supplementary file 1 [file animals-13-00348-s001.zip › Supplementary Table S4.pdf]

Supplementary Table S4 Statistics of sample sequencing data processing results.

Sample ID	Raw Reads	Clean Reads	Effective Reads	AvgLen (bp)	GC (%)	Q20 (%)	Q30 (%)	Effective (%)
W105d1	79770	79529	78179	418	53.89	99.17	96.47	98.01
W105d7	79895	79665	77587	417	54.27	99.14	96.4	97.11
W105d8	80193	79971	78046	418	53.96	99.15	96.42	97.32
W105d9	80002	79792	78538	420	54.45	99.13	96.35	98.17
W105d10	79900	79672	77766	418	53.72	99.13	96.32	97.33
W105d11	80028	79741	78378	418	54.02	99.12	96.3	97.94
W105d12	80244	80007	78118	417	54	99.16	96.45	97.35
W105d2	79650	79422	77819	419	53.73	99.16	96.43	97.7
W105d3	79644	79437	76681	418	54.11	99.16	96.44	96.28
W105d4	80096	79866	78227	419	53.87	99.18	96.51	97.67
W105d5	80268	79881	77970	420	55.26	99.08	96.2	97.14
W105d6	80215	79976	78095	419	54.04	99.15	96.38	97.36
W133d1	80171	79937	77819	418	53.68	99.15	96.41	97.07
W133d7	79890	79681	77801	420	53.42	99.13	96.31	97.39
W133d8	79904	79699	77900	419	53.95	99.16	96.44	97.49
W133d9	79994	79755	77517	420	54.18	99.17	96.43	96.9
W133d10	79949	79717	77736	418	53.78	99.19	96.52	97.23
W133d11	79919	79685	77385	419	53.89	99.18	96.51	96.83
W133d12	79839	79604	77863	418	53.54	99.19	96.53	97.53
W133d2	80283	80045	78353	419	53.55	99.13	96.34	97.6
W133d3	80057	79838	78224	419	53.52	99.15	96.42	97.71
W133d4	79846	79615	76031	419	52.98	99.13	96.36	95.22
W133d5	80026	79791	78015	419	54.11	99.13	96.32	97.49
W133d6	79757	79541	77297	418	53.05	99.15	96.4	96.92
W161d1	79914	79662	78851	417	53.3	99.19	96.5	98.67
W161d9	80142	79893	78208	418	53.46	99.16	96.45	97.59
W161d10	79779	79563	77681	419	53.09	99.14	96.36	97.37
W161d11	79850	79604	77757	418	53.78	99.15	96.39	97.38
W161d12	79939	79703	78190	418	53.69	99.18	96.5	97.81
W161d2	79987	79786	78170	418	53.21	99.16	96.4	97.73
W161d3	79972	79742	78166	419	53.58	99.17	96.41	97.74
W161d4	79971	79773	76145	416	53.9	99.2	96.54	95.22
W161d5	80073	79843	78177	417	53.32	99.19	96.51	97.63
W161d6	79929	79702	78043	418	53.13	99.14	96.37	97.64
W161d7	80031	79827	77863	418	53.04	99.17	96.48	97.29
W161d8	80216	79987	78006	416	53.62	99.17	96.48	97.24
W1d1	80013	79801	75764	416	54.39	99.2	96.57	94.69
W1d7	80146	79920	79455	424	54.81	99.22	96.56	99.14
W1d8	80030	79807	76378	421	52.82	99.2	96.51	95.44
W1d9	79929	79720	78686	417	55.92	99.21	96.59	98.44

W1d10	80204	79944	76279	414	54.08	99.23	96.65	95.11
W1d11	79918	79736	79358	428	55.07	99.2	96.48	99.3
W1d2	79684	79430	76650	418	55.82	99.2	96.58	96.19
W1d12	80064	79804	74819	417	54.12	99.19	96.54	93.45
W1d3	79985	79754	75981	415	54.02	99.22	96.66	94.99
W1d4	80593	80392	72509	417	54.78	99.22	96.63	89.97
W1d5	80417	80202	75190	419	54.48	99.18	96.48	93.5
W1d6	79947	79722	76275	419	55.41	99.16	96.43	95.41
W27d1	80212	80013	77540	417	52.71	99.19	96.48	96.67
W27d8	80132	79931	74288	417	53.89	99.21	96.6	92.71
W27d9	79980	79757	76204	419	53.24	99.15	96.39	95.28
W27d10	79945	79728	76839	416	51.91	99.15	96.41	96.11
W27d11	80410	80178	77951	415	51.9	99.21	96.59	96.94
W27d2	80143	79930	78020	418	52.26	99.13	96.31	97.35
W27d3	79877	79656	77466	416	51.98	99.16	96.4	96.98
W27d4	79769	79570	72537	417	52.47	99.17	96.44	90.93
W27d5	79775	79566	75049	418	52.56	99.15	96.41	94.08
W27d6	80115	79891	77358	417	52.66	99.18	96.48	96.56
W27d7	80094	79871	77214	418	52.29	99.18	96.53	96.4
W53d6	80245	79992	78107	416	52.99	99.17	96.49	97.34
W53d7	79862	79628	77385	417	53.44	99.18	96.51	96.9
W53d8	80211	80008	77855	418	53.35	99.2	96.57	97.06
W53d9	80108	79877	77116	418	52.72	99.17	96.45	96.27
W53d10	80072	79833	77407	418	52.7	99.15	96.37	96.67
W53d11	79850	79660	77732	415	52.63	99.21	96.63	97.35
W53d12	79918	79666	77176	417	52.96	99.16	96.47	96.57
W53d1	79967	79753	77938	417	53.24	99.21	96.59	97.46
W53d2	79388	79144	77375	418	53.61	99.16	96.45	97.46
W53d3	79893	79688	77865	415	52.83	99.22	96.65	97.46
W53d4	80410	80193	77363	416	53.1	99.24	96.7	96.21
W53d5	79872	79663	77827	416	53.29	99.21	96.62	97.44
