# Supplementary figures and images for: Dynamic Changes in the Gut Microbiota and Metabolites during the Growth of Hainan Wenchang Chickens
Source: Animals (Basel). 2023 Jan 19;13(3):348. doi: 10.3390/ani13030348 (PMC9913245; doi:10.3390/ani13030348)

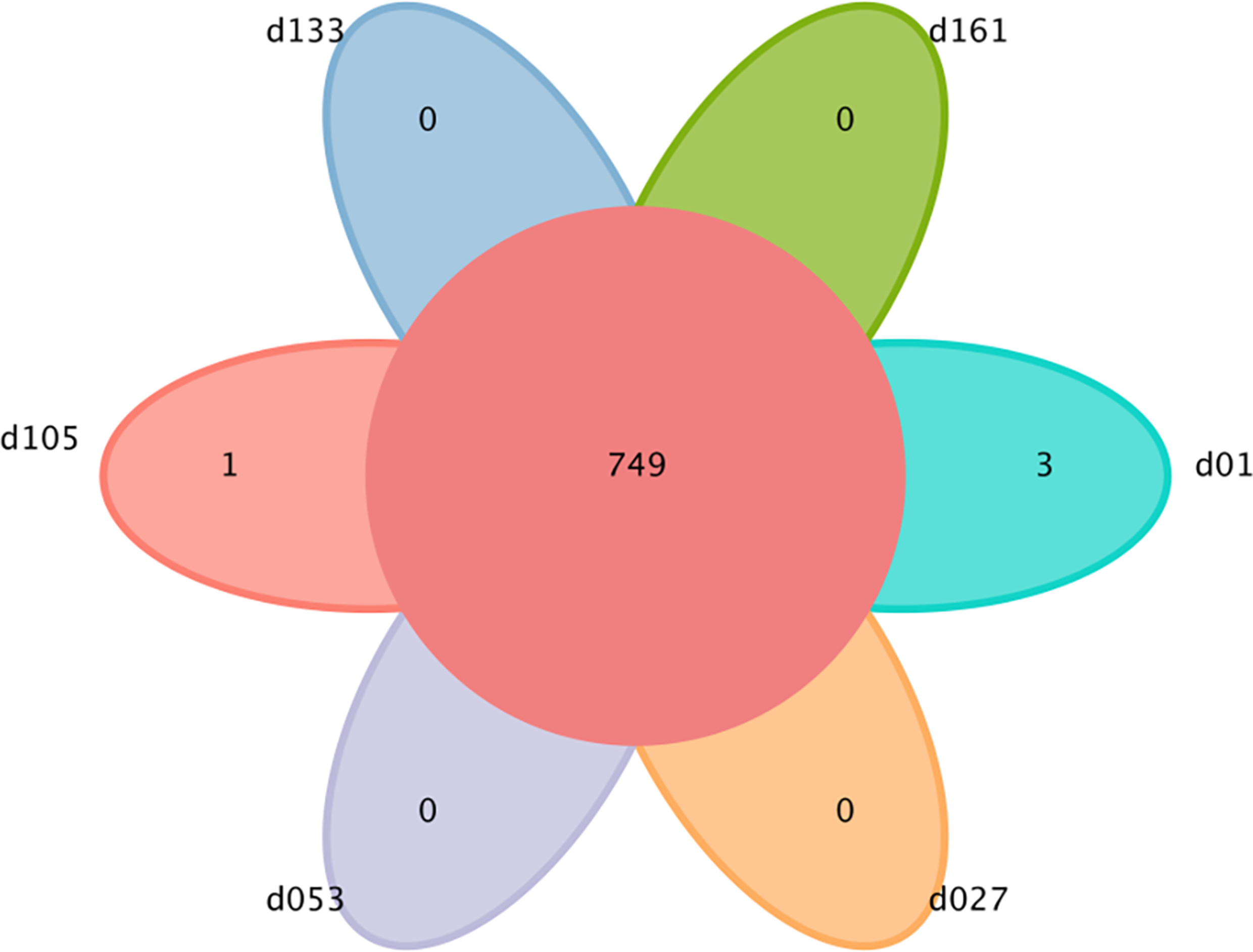

Supplement: Supplementary file 1 [file animals-13-00348-s001.zip › Supplementary Figure S1.tif]

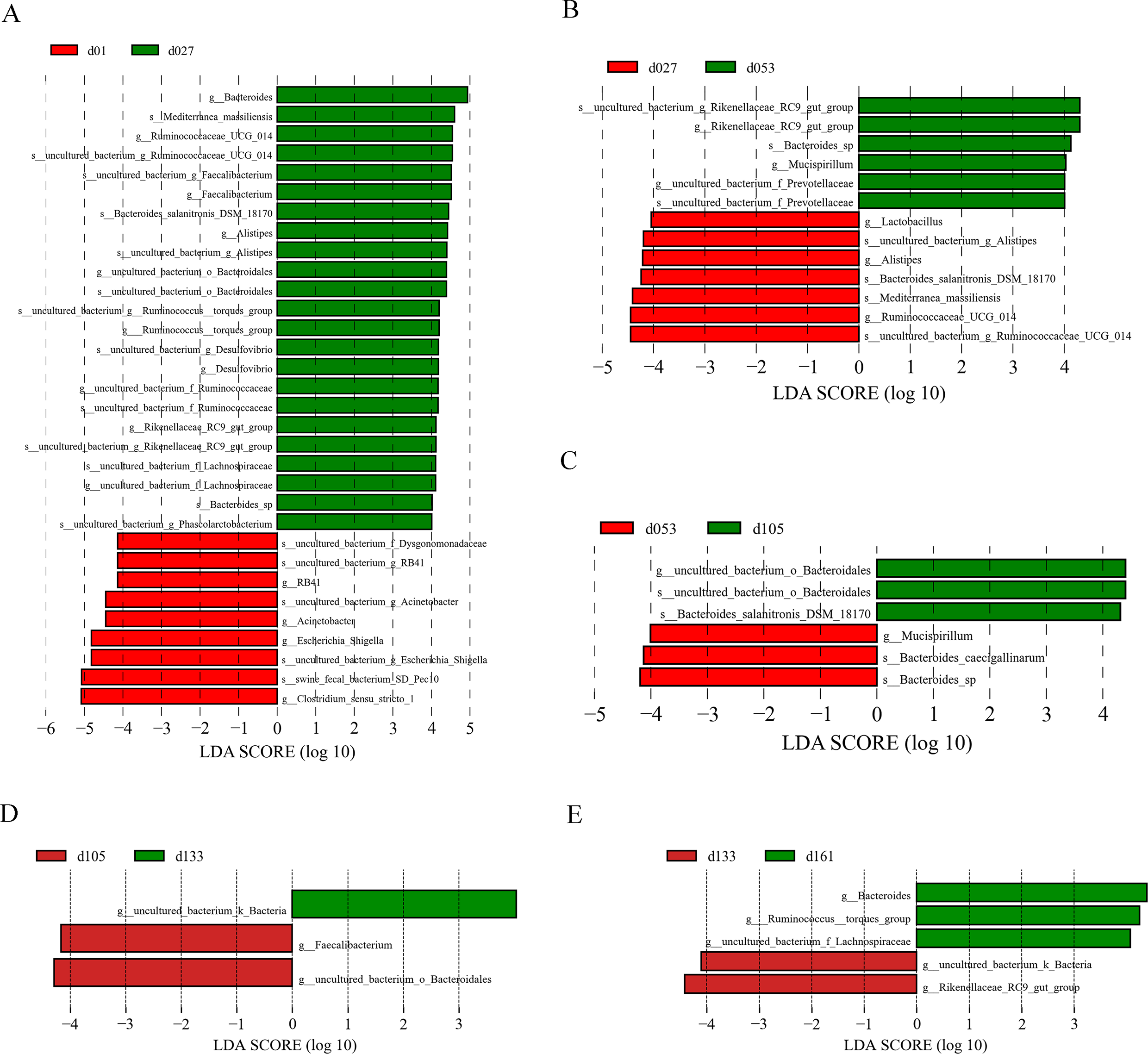

Supplement: Supplementary file 1 [file animals-13-00348-s001.zip › Supplementary Figure S2.tif]

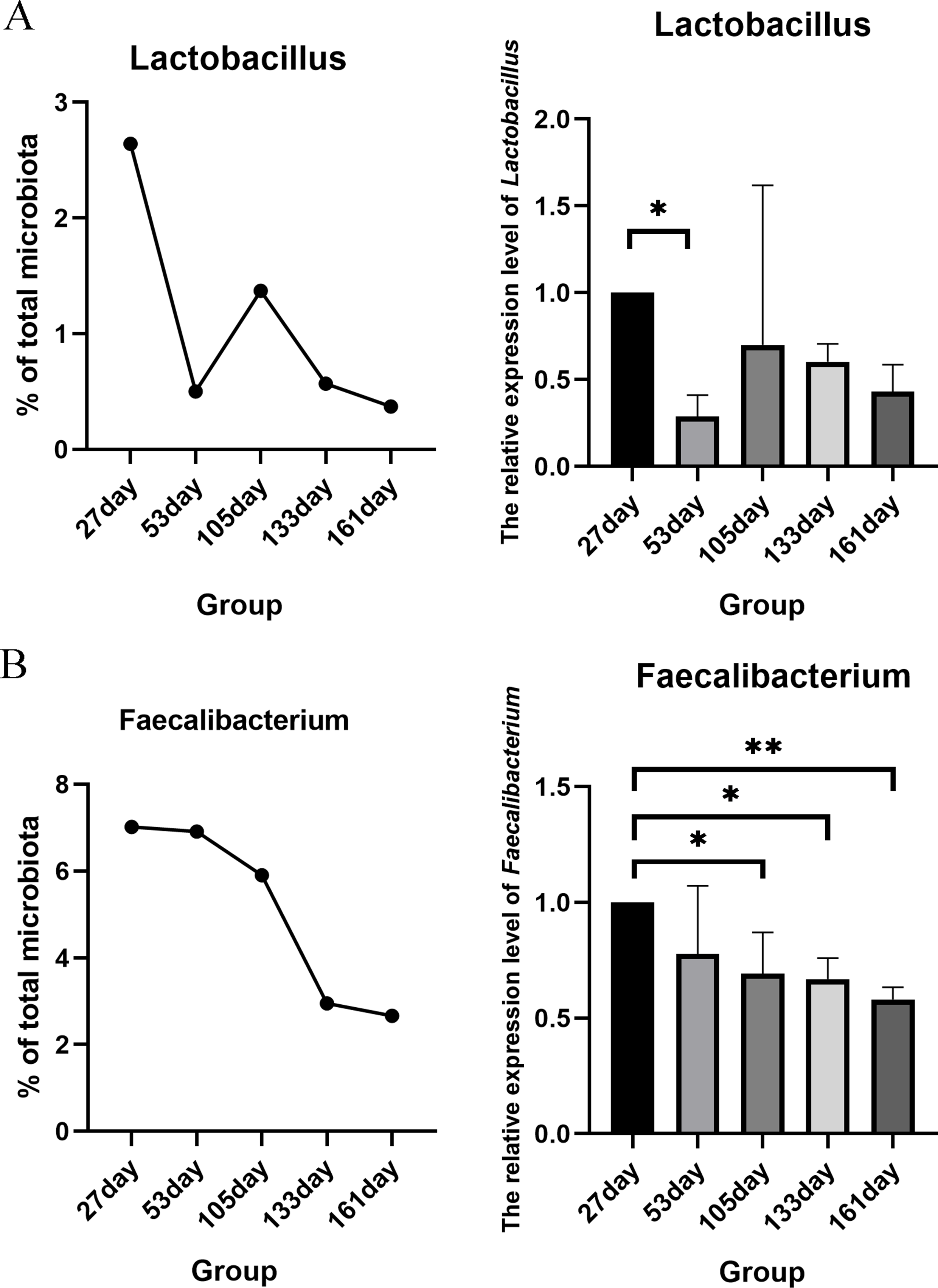

Supplement: Supplementary file 1 [file animals-13-00348-s001.zip › Supplementary Figure S3.tif]

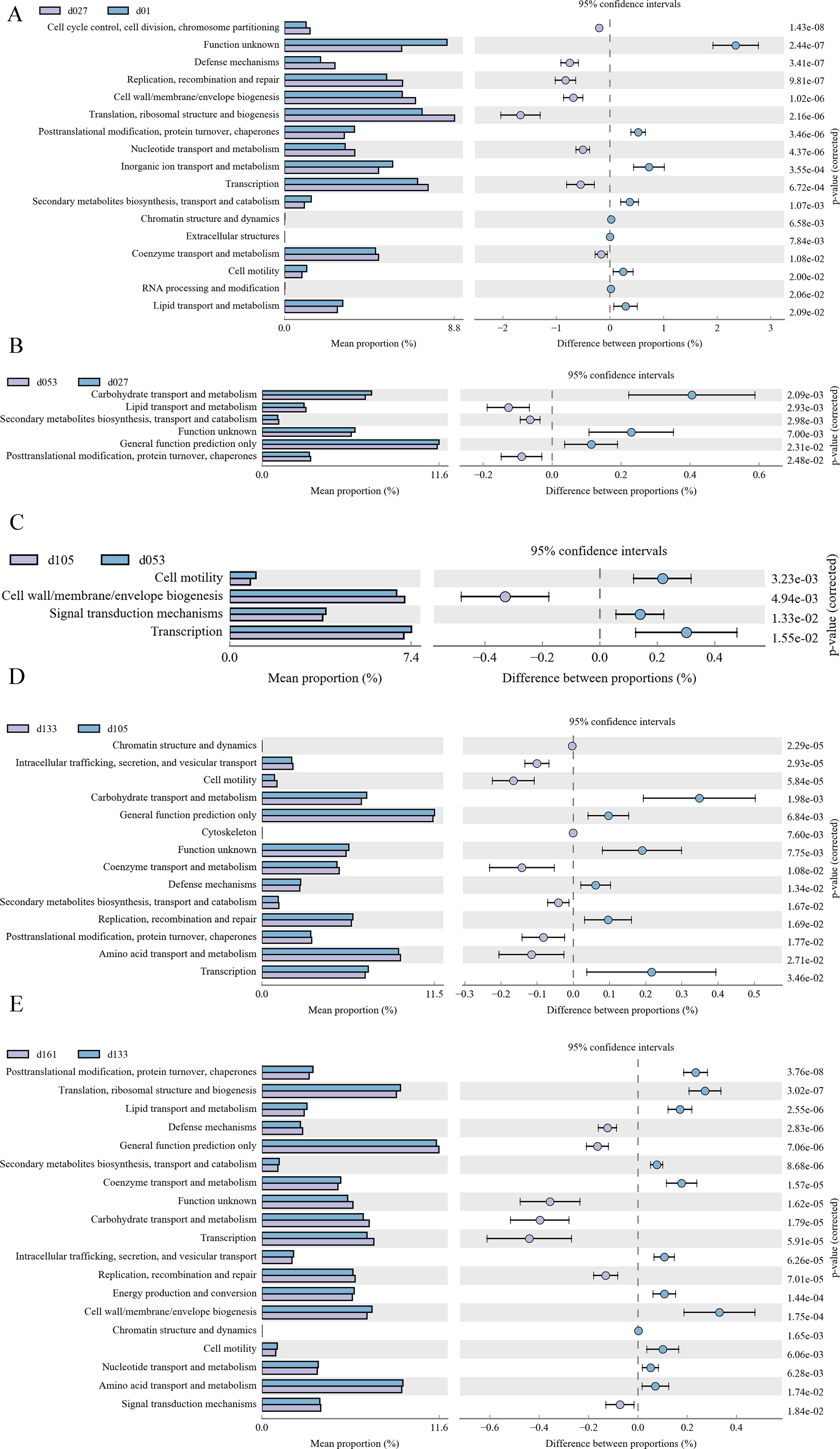

Supplement: Supplementary file 1 [file animals-13-00348-s001.zip › Supplementary Figure S4.tif]

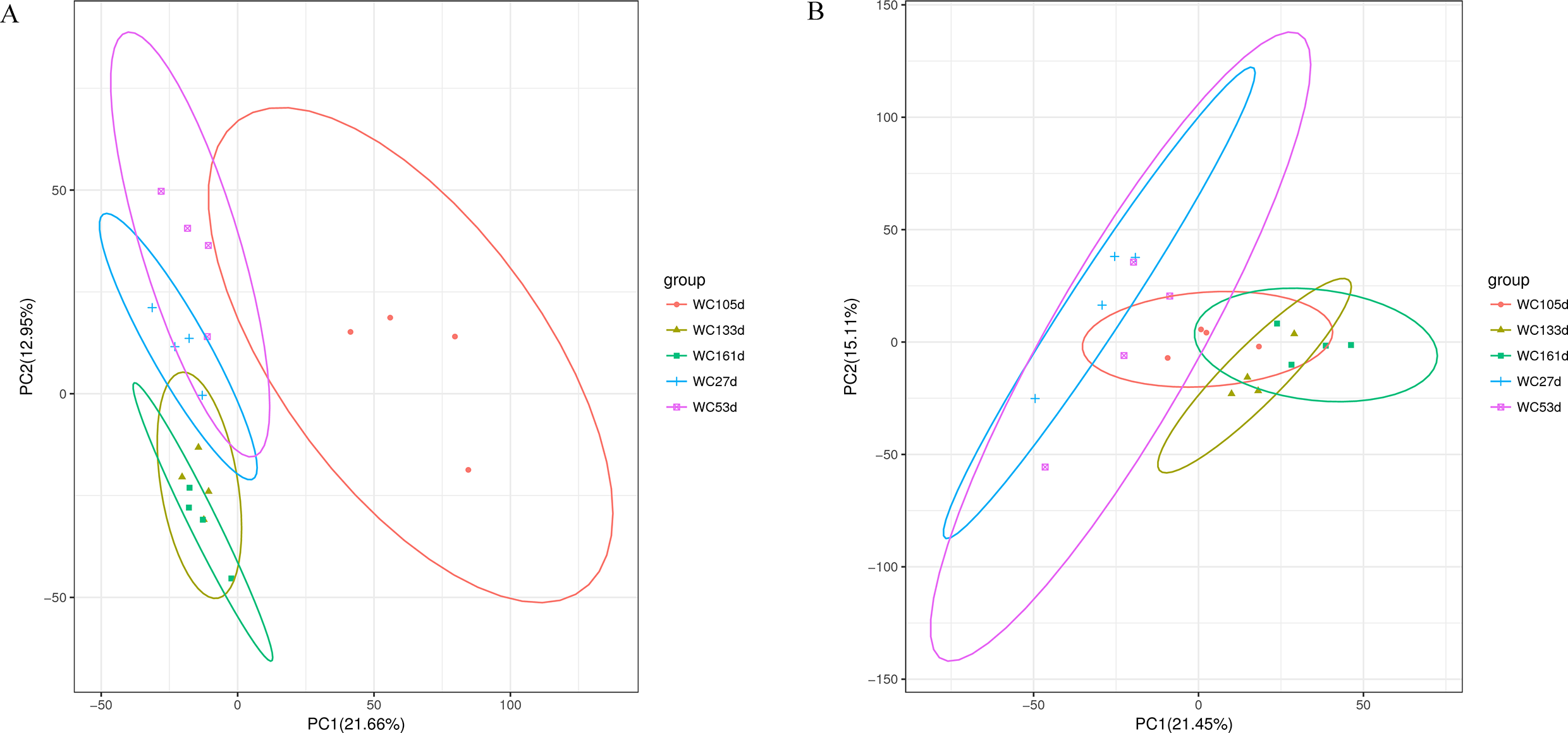

Supplement: Supplementary file 1 [file animals-13-00348-s001.zip › Supplementary Figure S5.tif]
